# Can deepfakes manipulate us? Assessing the evidence via a critical scoping review

**DOI:** 10.1371/journal.pone.0320124

**Published:** 2025-05-02

**Authors:** Didier Ching, John Twomey, Matthew P. Aylett, Michael Quayle, Conor Linehan, Gillian Murphy

**Affiliations:** 1 School of Applied Psychology, University College Cork, Cork, Ireland; 2 Lero the Research Ireland Centre for Software, Limerick, Ireland; 3 CereProc Ltd, Edinburgh, United Kingdom; 4 University of Heriot Watt, Edinburgh, United Kingdom; 5 Centre for Social Issues Research and Department of Psychology, University of Limerick, Limerick, Ireland; 6 Department of Psychology, School of Applied Human Sciences, University of KwaZulu-Natal, Pietermaritzburg, South Africa; Tianjin University, CHINA

## Abstract

Deepfakes are one of the most recent developments in misinformation technology and are capable of superimposing one person’s face onto another in video format. The potential of this technology to defame and cause harm is clear. However, despite the grave concerns expressed about deepfakes, these concerns are rarely accompanied with empirical evidence. We present a scoping review of the existing empirical studies that aim to investigate the effects of viewing deepfakes on people’s beliefs, memories, and behaviour. Five databases were searched, producing an initial sample of 2004 papers, from which 22 relevant papers were identified, varying in methodology and research methods used. Overall, we found that the early studies on this topic have often produced inconclusive findings regarding the existence of uniquely persuasive or convincing effects of deepfake exposure. Moreover, many experiments demonstrated poor methodology and did not include a non-deepfake comparator (e.g., text-based misinformation). We conclude that speculation and scare mongering about dystopian uses of deepfake technologies has far outpaced experimental research that assess these harms. We close by offering insights on how to conduct improved empirical work in this area.

## Introduction

Misinformation can be defined as information that is inaccurate or untrue, and which is unintentionally disseminated [1], whereas intentionally disseminated inaccurate information is defined as disinformation [2]. We will be using the term “misinformation” as shorthand for inaccurate information disseminated regardless of intent in this paper. Research into misinformation in the form of fake news articles, misleading health articles, and doctored photos shows that attitudes, behaviours, and memories can be manipulated through these media [3–6]. “Deepfakes” are one type of media that has been used to spread misinformation and has raised serious concerns among the public and academic community [7].

Deepfakes are synthetic media generated using AI neural network technology [8]. Commonly, deepfakes are presented as manipulated videos showing one person’s face superimposed onto another person effectively creating a video of a person doing something they did not do. Deepfakes can potentially have exciting and positive applications, such as translating educational materials or films [9,10], in films and music videos for entertainment purposes [11,12], or even in museums to resurrect historical figures [13]. However, concern about the negative applications of this technology such as defamation, revenge pornography, and political sabotage [7] often overshadows other applications.

Many authors have speculated about the potential threats of deepfakes and the behavioural, political, and legal implications of this technology [14–17]. For example, Chesney and Citron [18] have suggested that deepfakes could destabilise society by instilling mistrust in citizens towards all media and influence voting behaviours thus jeopardizing election integrity. Most of the academic papers on this topic are speculative or essay-based rather than empirical, such that there is a wealth of literature decrying the potential harms caused by deepfakes, but a relatively small number of empirical papers that investigate evidence for these harms [7,14,15,19]. A review of existing evidence is necessary to consolidate what we currently know about the effects of deepfake exposure in the face of so much speculation. In this paper, we present a scoping review mapping the current state of deepfake literature regarding the effects of viewing deepfakes on people’s beliefs, memories, and behaviour. These outcomes were chosen to reflect elements of human psychology which are vulnerable to deepfake manipulation according to non-empirical papers and discussions [7,9,18]. We will also be analysing the types of deepfakes used in empirical studies to identify strengths and weaknesses of their study design and methodology. This is to generate recommendations for future empirical study design.

While traditionally, scoping reviews do not aim to critically appraise the included studies, we will be doing so for several reasons. Firstly, since preliminary searches indicate a small amount of relevant empirical deepfake literature, critically appraising the available literature will yield a more in-depth and accurate analysis of the direction of the literature. Our research questions are concerned with whether evidence exists to suggest that deepfake exposure has a measurable effect on an individual’s beliefs, memories, and/or behaviours. Appraising the evidence of existing research studies is crucial because it helps us accurately identify high quality research which contribute as evidence to answering our research questions. Surface-level analysis of study findings in our sample will include papers which could possibly give us a false representation of the strength of evidence in particular research areas.

This scoping review aims to answer four questions: RQ1) Is there evidence to suggest that deepfake exposure has measurable impacts on beliefs, memories, or behaviours of viewers? RQ2) Is there evidence to suggest that the effects of viewing deepfakes on beliefs, memories, and behaviours are different from those seen with misinformation spread via other media? RQ3) Is there evidence of social, psychological, and technical factors that influence the effects deepfakes have on beliefs, memory, and behaviour? RQ4) What kind of deepfake technology is being used in modern empirical deepfake research?

### Literature review

#### The threat of deepfakes.

There is an abundance of literature speculating about the harms that deepfakes could wreak upon society. For example, Albahar and Almalki [7] suggest that deepfakes could make fake news and misinformation more widely present in all forms of media, leading to citizens being unable to differentiate between real and misleading information. Indeed, some research has shown that individuals cannot discern real videos from deepfakes at a rate better than chance [20]. Just as worryingly, Kobis et al. [20] also showed that individuals overestimate their ability to discern deepfakes from real videos, possibly further increasing their susceptibility to deepfake deception. In a legal context, the validity of images and videos for use as evidence in courtrooms may be in jeopardy because video evidence, which is often considered the gold standard for proof, can no longer be trusted. In a political context, deepfakes could be used as a form of sabotage and defame candidates from opposing parties as you could create a deepfake depicting the opposition candidate saying something derogatory. These concerns are repeatedly highlighted in published literature [14,17,19,21]. Chesney and Citron [18] further discuss that deepfake technology could distort democratic discourse, manipulate elections, and undermine public safety. Concerns around deepfakes have also spread to military and national security risks where, according to Chesney and Citron [18], deepfakes “have utility as a form of disinformation supporting strategic, operational, or even tactical deception” (p. 1783).

Deepfakes may also cause harm in other ways. Firstly, some authors argue that deepfakes could give rise to a “Liar’s Dividend”, where factual information from media and news in society is invalidated and incorrectly labelled as fake news [18]. As an example, a politician could deny the veracity of an incriminating video of themselves by declaring it as a deepfake. This could serve to undermine citizen’s trust in any information they receive regardless of authenticity. Similarly, deepfakes may cause “epistemic pollution” where knowledge of the existence of deepfakes may cause individuals to accuse real videos of being deepfakes [14]. For example, Twomey et al. [22] explored discourses surrounding the Russo-Ukrainian war on Twitter. Surprisingly, the most frequent form of deepfake-related misinformation found in the dataset was real media being labelled as being deepfake Secondly, deepfakes may cause reputational harm, even in situations where the videos have been identified as inauthentic, such as with the generation of deepfake revenge pornography [23]. The reputational damage comes from being depicted in realistic-looking sexually suggestive positions, meant to embarrass and humiliate the victims [23,24]. Importantly, the warnings seen so frequently in literature about deepfakes are based on the assumption that deepfakes can successfully influence the beliefs, memories, and behaviours of viewers. However, there is no existing review that assembles and synthesises the available evidence, to determine whether that view is supported empirically.

#### Misinformation effects on beliefs, memories, and behaviour.

Decades of research have demonstrated that encountering misinformation can affect people’s beliefs, with more recent research focused on fake news stories. Exposure to fake news is correlated with a range of adverse outcomes. For example, Ognyanova et al. [25] demonstrated that false information is linked to reduced trust in mainstream media, while Balmas [26] showed that subjection to political misinformation is associated with attitudes of inefficacy, alienation, and cynicism towards political candidates. Misinformation in a health context can also influence people’s beliefs. For example, studies have shown that misinformation about COVID-19 during the pandemic has been associated with increased vaccine hesitancy [27–29]. Beyond text-based fake news stories, research has shown that doctored photographs and other forms of manipulated media can affect beliefs as well. For example, Hameleers et al. [30] demonstrated that a combination of doctored photos and misinformation texts was more credible than text alone, suggesting that visual media could enhance the potency of misinformation. In agreement with this finding, Nash et al. [31], discovered a small but consistent effect of doctored photos on people’s beliefs about prior events, as well as the potential for doctored videos to distort participants’ beliefs about actions they had performed [32,33]. Messaris & Abraham [34] suggests that visual information provides an evidential quality that enhances its credibility over text. The multi-modal audio-visual aspect of deepfakes may suggest an enhanced potency at generating and propagating misinformation. Current literature has also supported this sentiment, with researchers speculating that the visual aspect of deepfakes makes them more likely to affect our beliefs [35,36].

A large body of evidence has also demonstrated clear effects of misinformation on memory. Elizabeth Loftus first established “the misinformation effect” where post-event misleading information can alter one’s memories of an event [37]. The misinformation effect can be seen in political settings where false memories were induced by fake news stories for the abortion referendum in Ireland [38] and Brexit [39]. Similarly, there is also evidence to support the distortion of memory due to exposure to doctored photos. Wade et al. [40] induced false memories of a childhood hot air balloon ride by showing participants doctored photos. This effect was replicated by Sacchi et al. [41] when they found that doctored photos caused participants to misremember elements of important historical events such as the 1989 Tiananmen Square protest in Beijing and the 2003 Rome anti-war protest. Many researchers have proposed that deepfakes will similarly induce vivid false memories [35,36,42]. However, Greene et al. [43] and Garry and Wade [44] found that visual-based misinformation in the form of photographs did not induce false memories better than simple text narratives. They reasoned that the narratives encouraged deeper processing of misinformation, leading to more significant distortions in memory compared to photographs. Many previous studies, including Wade et al. [40] and Saachi [41] did not include text-based misinformation as a comparator, so though they reported substantial rates of false memories, these may not have been due to the doctored images. Thus, it is difficult to predict whether deepfakes might be more potent at manipulating memory than non-technical means of conveying misinformation.

There are significantly fewer studies on the effects of misinformation on behaviours. Research on the COVID-19 pandemic has shown that misinformation exposure from social media has induced a decline in intent to be vaccinated [45]. Similarly, a decline in childhood vaccination rates against measles, mumps, and rubella (MMR) was found to be related to misinformation about the MMR vaccine [46]. Greene and Murphy [3] experimentally quantified the effects of misinformation exposure on behavioural outcomes and found a small but significant effect of COVID-19 misinformation on behavioural intentions. Conversely, de Saint Laurent et al. [29] found no effect of COVID-19 misinformation on behavioural intentions. The existing literature focuses on health contexts and misinformation delivered through text only with little agreement, thus it is unclear how misinformation affects behavioural intentions in different contexts and mediums. However, general claims have been made in the literature about the ability of misinformation to modify and manipulate behaviour, threaten individual autonomy, and jeopardize democracy [47].

#### Factors influencing the effects of misinformation on people’s beliefs, memories, and behaviour.

There is a wealth of research investigating the individual factors that lead people to be influenced by inaccurate or false information. For example, people who engage in analytical reasoning and reflection are less likely to be deceived by misinformation [48,49]. Pennycook and Rand [5] and Bronstein et al. [50] also mirror these findings, showing the negative correlation between cognitive reasoning and misinformation susceptibility. This shows that the tendency to engage in careful analysis rather than the intuitive acceptance of information is important in discerning truth from misinformation. This idea has then been used to design interventions to help combat misinformation. For example, misinformation “inoculation” or “pre-bunking” is a strategy which forewarns individuals of misinformation by providing accurate information on the misinformation content they are about to view. This strategy works by provoking individuals to critically analyse the content they are about to view, bypassing simple intuitive acceptance of information in favour of a purposeful engagement with the information [51]. Despite the wealth of literature investigating these individual factors and their implications, very few of these studies have specifically investigated deepfakes. Due to the multi-modal format of deepfakes, it is not known whether deepfakes are more, less, or equally effective in delivering misinformation and manipulating people’s beliefs, memories, and behaviours compared to misinformation presented as text or photos, despite many claiming this to be the case [35,36]. Also, it is evident that factors such as cognitive style can influence the effects of misinformation on beliefs, memory, and behaviour and it would be reasonable to assume that deepfake misinformation would follow closely with this literature. The current scoping review will provide a clearer picture regarding the impact of deepfakes. be the first step to establishing the effects of deepfakes in misinformation literature.

## Materials and methods

### Eligibility criteria

In order to be included in the review, research papers were required to either test the effect of viewing deepfakes on beliefs, memories, or behaviour, compare the effects of deepfakes with other pre-existing forms of misinformation, or identify factors influencing the effects of viewing deepfakes. Papers were required to be published in peer-reviewed journals and written in English. The period of literature included spanned January 2017 to April 2024 (when we conducted our search). Years 2017–2024 were chosen as the first recorded incident of deepfakes occurred in 2017 [52]. Any form of empirical study meeting these criteria were included, such as quantitative, qualitative, or mixed methods approaches.

### Information sources

5 electronic databases were searched on April 13^th^, 2024: PsycINFO (through the platform of American Psychological Association), Web of Science Core Collection, ACM, DBLP, and Scopus.

### Search strategy

The five electronic databases were searched using the terms: [deepfake] AND [Belief OR Memory OR Behavior OR Attitude]. The keywords were purposefully broad with no limitations to keep a wide scope due to the expected low number of deepfake psychological studies. Other keywords were considered such as “trust” or “believe” but these terms were considered to be already covered within the parameters of our search strategy. At peer-review stage, we re-conducted the search with these terms included, but did not find any further relevant papers. The search terms “belief”, “memory” “behavior” and “attitude” were considered sufficiently broad to cover relevant forms of manipulation. As well as this, due to the possible broad interpretation of deepfakes in research papers [53], we considered including other terms to describe AI-generated videos or deepfake software, such as “AI generated videos” or “Fake Videos”, but practically, it was difficult to make this list exhaustive without including largely irrelevant general search terms (such as “ AI generated”) which would yield a redundant amount of papers. The references of relevant papers were also hand-searched for studies that fit the eligibility criteria.

### Screening process

**Fig 1:**
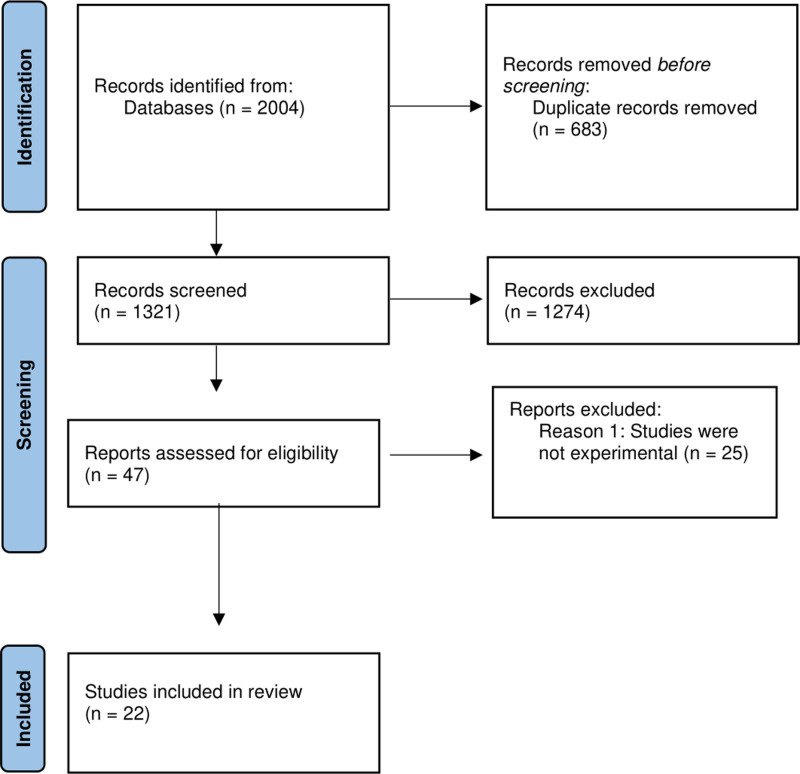
Flow chart of screening process.

The research papers were screened by two reviewers, using the Rayyan software package [54]. Titles and abstracts were screened first and papers that did not include an empirical element were excluded. Subsequently, a full-text screening process was carried out on the remaining papers screening out papers that 1) did not directly measure the effects of viewing deepfakes on beliefs, memories, or behaviours, 2) compare the effects of deepfakes to other forms of misinformation, or 3) investigate potential psychological, social, or technical factors which influences the effect of deepfakes of an individual’s beliefs, memories, and/or behaviours. Inter-rater agreement was 80%, and disagreements were resolved by discussion. Following the title and abstract screening and the full-text screening, a total of 22 papers met the inclusion criteria and were included in the review. The selection and screening process details is illustrated in the PRISMA flow diagram in Fig 1.

### Extraction and analysis

Following guidance from Arksey and O’Malley’s [55] framework for conducting scoping reviews, data from the included articles for the review are charted below in Table 1. This data includes each paper’s research question and main findings, as well as extracted data such as study design, source of deepfake, comparisons, and outcome measures. A narrative synthesis was carried out to collate all the data together to answer the four research questions.

**Table 1:** Descriptive data of all included studies.

Author	Descriptives						
	Year	Study Design	Source of deepfake	Comparisons	Methodology	Outcome Measures	Delay between exposure and outcome
Ahmed	2021a	Survey	N/A	None	Survey	Inadvertent deepfake sharing, political interest, cognitive ability, social network size, political trust, deepfake exposure and deepfake concern	None
Ahmed	2021b	Survey	N/A	None	Survey	Social media news use, deepfake concern, deepfake exposure, inadvertent deepfake sharing, and cognitive ability	None
Ahmed	2021c	Experimental	Canny AI art installation	None	Participants were exposed to a deepfake with an educational caption or no captions and asked to answer questions	Perceived claim accuracy, sharing intention, and cognitive ability	None
Ahmed, Wang, & Bee	2023	Experimental	Canny AI art installation	None	Participants asked to rate news article as real or fake twice. Experimental group sees a deepfake in-between ratings.	Perceived accuracy of stimuli and cognitive ability	None
Ahmed, Ting Ng, & Bee	2023	Experimental Survey	Canny AI art installation	None	Survey	Perceived accuracy, Self-regulation, Fear of missing out, cognitive ability, and deepfake sharing	None
Ahmed & Chua	2023	Experimental Survey	RepresentUS	Deepfakes vs cheapfakes vs audio	Survey. Participants view misinformation in different modalities (deepfakes vs cheapfakes vs audio) and asked to answer questions.	Perceived accuracy, sharing intentions, and cognitive ability	None
							None
Dobber, Metoui, Trilling, Helberger, & de Vreese	2020	Experimental	Author-made deepfake using Tacotron 2 to synthesise voice and AI based lip synchronizing Technique	None	Participants exposed to political deepfake in experimental condition and asked to answer questions.	Attitudes towards politician, attitude towards politician’s party, and ethics	None
Hameleers, van der Meer, & Dobber	2022	Experimental	VFX and Deepfake artist	Text-only to deepfake	Participants exposed to political deepfake in experimental condition and asked to answer questions	Issue agreement, credibility, and evaluations of the depicted politician	None
Hameleers, van der Meer, & Dobber	2024	Experimental	VFX and Deepfake artist	None	Participants exposed to political deepfake/text misinformation in experimental condition and asked to answer questions	Perceived credibility, delegitimizing effect on evaluations of political actor	None
Hameleers	2024	Experimental	Collaboration with computer vision and AI-Experts. Professional voice actor for speech	Deepfakes vs cheapfakes vs text	Participants exposed to political deepfake/cheapfake in experimental condition and asked to answer questions	Perceived credibility	None
Murphy, Ching, Twomey, & Linehan	2023	Experimental	YouTube	Deepfake vs text	Participants exposed to deepfakes in films and asked if they remembered seeing the film.	Presence of false memories	None
Lu & Chu	2023	Experimental	“Preface” mobile app	None	Participants exposed to deepfakes of people they were told to be deceased and asked to answer questions.	Perceived realism, identification, compassion, perceived desecration of the dead, surprise, policy support, and activism intentions	None
Sharma, Jain, Behl, Baabdullah, Giannakis, Dwivedi	2023	Experimental Survey	Custom created by deep learning technology professional	None	Participants exposed to political deepfakes and asked to answer questions as part of a survey.	Intentions to share, intention to verify political deepfake video, and moral consciousness	None
Yu-Leung Ng	2023	Experimental Survey	Developed by French charity Solidarite Sida and computer scientists	None	Participants exposed to political deepfakes/Real political videos and asked to answer questions as part of a survey. Before each video, there was either a description explaining what deepfakes are or no description.	Perceived video fakeness, perceived message fakeness, perceived dangerousness, and perceived trustworthiness	None
Jin, Zhang, Gao, Gao, Zhou, Yu, & Wang	2023	Experimental Survey	YouTube	None	Participants were exposed to deepfake videos and asked to answer questions as part of a survey. The videos were made to emulate real videos on platforms which included the number of followers, popularity, description as well as definition, duration, and editing.	Credibility, realism, source credibility, and popularity	None
Hwang, Ryu, & Jeong	2021	Experimental	Canny AI art installation	Text-only condition to deepfake + text	Participants were exposed to one of three interventions: deepfake specific literacy education, general disinformation education, or no literacy education. Participants were then exposed to deepfake videos and asked to answer questions afterwards.	Vividness, persuasiveness, credibility, and intent to share	None
Iacobucci, De Cicco, Michetti, Palumbo, & Pagliaro	2021	Experimental Survey	YouTube	None	Participants were assigned to either read an excerpt on deepfakes or read nothing at all before viewing a deepfake video. Participants were then asked to answer questions afterwards.	Bullshit receptivity, attitude towards video, and intention to share video	None
Lee & Shin	2022a	Experimental	Faceswap Software	Text-only condition to text-photo to deepfakes	Participants were exposed to text, text-photo, or deepfake misinformation and asked to answer questions. Participants in a second experiment either saw a fake news correction or no correction before deepfakes and asked to answer questions afterwards.	Source vividness, fake news credibility, and fake news engagement intentions	None
Lee & Shin	2022b	Experimental	Faceswap Software	None	Participants were exposed to a deepfake and asked to answer questions afterwards.	News Credibility, Viral Behavioral Intention, Pre-existing Issue Attitude, Cost Perception, and Prior Source Familiarity	None
Murphy & Flynn	2021	Experimental	YouTube, Faceswap software, and Canny AI art installation	Text-only condition to text-photo to deepfakes	Participants were exposed to misinformation presented as text, text-photo, or deepfake and asked whether they remembered the contents of the misinformation happening.	Self-reported false memory and false memory vividness	None
Vaccari & Chadwick	2020	Experimental	YouTube	None	Participants were exposed to a deepfake video and asked to answer questions afterwards.	Participant’s belief in deepfake videos and trust in news and social media	None
Wu, Ma, & Zhang	2021	Experimental	ZAO deepfake app	None	Participants were exposed to a deepfake video and asked to answer questions afterwards.	Body image, state appearance self-esteem, state appearance comparison, and attractive possible self (APS) perception	None

## Results

Out of the 22 included papers, 14 studies answered RQ1, six studies answered RQ2, 13 studies answered RQ3, and all 22 studies contributed to answering RQ4. The total studies added up to more than 22 papers because some papers had properties which helped answer multiple research questions. (see Table 1).

### Descriptive data

#### RQ1. Is there evidence to suggest that deepfake exposure has a measurable impact on people’s beliefs, memories, or behaviour?.

Our review yielded eight studies that examined how deepfake exposure does have a measurable effect on people’s beliefs across four research topics. Firstly, experiments by Hwang et al. [56] and Lee and Shin [57] found that participants rated misinformation messages presented as a deepfake as more vivid and credible compared to text and text-photo formats, suggesting deepfakes may be a uniquely persuasive form of misinformation. Secondly, Hameleers et al. [58] and Dobber et al. [59] document the effects of deepfakes on beliefs in a political context by exposing participants to deepfakes of political figures saying disrespectful [59] and politically incongruent [58] statements. Their results show that deepfakes negatively impact people’s attitudes towards the depicted politicians, even if the deepfake was not rated as credible [58]. Thirdly, Vaccari and Chadwick [36] and Ahmed et al. [60] show that deepfakes can also be harmful by influencing our beliefs about authentic information, promoting mistrust in news and social media [36], as well as affecting our perceptions of real information about public figures by retroactively showing participants deepfakes of those public figures [60]. Finally, studies by Wu et al. [61] and Lu and Chu [62] investigated how deepfakes influence our beliefs in a potentially positive way. Wu et al. [61] demonstrated how viewing deepfakes of oneself on the body of a celebrity positively impacts young women’s feelings of satisfaction with their own attractiveness and self-image while Lu and Chu [62] explores the impact of so-called “digital resurrections” of deceased individuals and how they could be used advocate for issues related to their cause of death. However, they found that individuals considered these resurrections as disrespectful and demonstrated reduced support for these issues. While there is evidence that deepfake exposure has a measurable effect on beliefs, the varied research questions and relatively low number of studies in each research topic makes it difficult to identify obvious trends in the literature.

Two studies in the review investigated how deepfakes affect memory and potentially create false memories. Murphy and Flynn [4] found that deepfakes can indeed affect people’s memories of events by creating false memories for events that did not occur, but this effect does not seem to be significantly different than other formats of misinformation such as text-only or text with a photo. Similarly, Murphy et al. [63] found that deepfakes were capable of inducing false memories in participants in an entertainment context when this technology is used to replace the faces of actors in films with other famous actors. Interestingly, deepfakes were no more effective at inducing false memories than simple textual misinformation. This finding is tentative and based on just two studies, but it suggests that while deepfakes can manipulate memories, concerns about our memories being *uniquely* affected by the rise of deepfake misinformation [35] may be overblown.

Finally, we identified four studies that indirectly explored how attitudes and behavioural intentions were impacted by deepfakes. Lee and Shin [57] and Hwang et al. [56], found that people perceived deepfaked messages as more credible and vivid compared to other formats of misinformation, such as text-only and text-photos. They also found that this increase in vividness and credibility was associated with elevated levels of engagement and sharing intentions. Similarly, Ahmed [64] found that individuals are more likely to share deepfakes online when they deem the deepfake to be credible, and Ahmed and Chua [65] found that deepfakes, compared to cheapfakes or audio deepfakes, are especially likely to be shared. Noticeably absent from these few studies of deepfakes on behaviour are studies on a wider variety of behaviours beyond deepfake sharing (e.g., vaccine uptake, voting choice, etc.) and any studies assessing real-life behaviours or behaviours over a longer time period. This echoes gaps in misinformation research more generally and is not unique to deepfake research [29]. Evidently, the current empirical literature examining the effects of deepfakes on beliefs, memory, and behaviour is insufficient in number to draw strong conclusions. While the initial studies suggest a measurable effect of deepfakes on beliefs, memories, and behaviours, the relatively low number of studies, the varied research findings, inconsistent deepfake stimuli, and lack of behavioural research highlight many unanswered questions.

#### RQ2. Is there evidence to suggest that the effects of deepfakes on beliefs, memories, and behaviour are different from pre-existing forms of misinformation?.

As shown in Table 1, we found just six studies that compared the deceptive potential of deepfakes to other misinformation formats such as text or text-photos [4,56,57,63,66,67]. These six studies demonstrated mixed results. For example, Murphy and Flynn [4] and Murphy et al. [63] both compared misinformation presented as deepfakes and as text to participants and found that deepfakes did not produce significantly different effects than the text conditions in inducing false memories in participants. Hameleers et al. [66] and Hameleers [67] found that deepfakes were not perceived as more credible than misinformation presented as text. In direct contrast to this, Lee and Shin [57] and Hwang et al. [56] compared deepfakes to misinformation text and texts with photos and found that misinformation messages presented as deepfakes were rated as more credible and vivid. Thus, the very sparse evidence to date suggests that at least when we assess memory distortion or credibility, there is no clear advantage for deepfakes relative to existing forms of misinformation. Despite much speculation as to the grave threats posed by deepfake technology and how much more potent deepfake misinformation is relative to other formats [7,19,35], we simply do not have the data and sufficient agreement of evidence to support these claims.

#### RQ3. Is there evidence of social, psychological, or technical factors that influence the effects deepfakes have on beliefs, memories, and behaviour?.

13 studies provided evidence of psychological, social, or technical factors which influence how deepfake exposure affects our beliefs, memories, and behaviours. Seven of these studies highlighted cognitive ability or a factor related to cognition as a factor that may influence the impact of deepfake exposure. Most of these studies considered cognitive ability as the ability to think analytically and be able to critically assess information or stimulus past simple intuition [5]. Ahmed [68], Ahmed [69], Ahmed et al. [70], and Ahmed and Chua [65] reported that individuals with higher cognitive ability were less likely to self-report the inadvertent sharing of deepfakes. This was measured by directly asking participants whether they had recently shared a deepfake and later discovered that the video was a hoax. This is problematic as it relies on participants’ own subjective interpretation of what deepfakes are and does not allow the reporting of deepfake sharing that went unnoticed. Individuals with higher levels of cognitive ability are less likely to consider deepfakes they see as credible or accurate [58,60] Finally, Iacobucci et al. [71] found that individuals with low levels of bullshit receptivity (the tendency of an individual to believe baseless claims) and primed with knowledge about deepfakes were more likely to recognise that a video was a deepfake. This experiment measured deepfake recognition by asking participants whether a clip of a face-swapped actor in the movie “The Shining” was similar to the original scene due to the actor’s acting ability, or digital video-editing technology.

Five studies found that an individual’s political interests, views of politicians, and ideology can also affect how deepfakes are interpreted and consumed [66,68,72–74]. Hameleers et al. [66] found that deepfake disinformation that was ideologically congruent was perceived as more credible than disinformation that was ideologically incongruent – however this was equally true for text-based disinformation, so does not suggest anything unique to deepfakes. Sharma et al. [74] found that political brand hate (an individual’s hatred towards a political party or politician due to who or what they represent) was associated with sharing deepfakes which represent the ideologically incongruent party or “brand” in poor light. Similarly, Lee and Shin [72] found that individuals were more likely to believe and intend to share deepfake news which advocated for positions congruent with their own beliefs than individuals with incongruent beliefs. Regardless of political stance or position agreement, Ahmed [68] reported that individuals who simply have an interest in politics are more likely to inadvertently share any deepfakes they encounter. However, this finding is unreliable due to the aforementioned issues of interpretation, self-reporting, and measurement of deepfake sharing behaviours. On a separate note, Ng [73] found that an individuals’ susceptibility to deepfake videos of a politician depends on the perceived trustworthiness and dangerousness of that politician. If an individual perceived a politician to be dangerous, then they are less likely to regard deepfake videos of them as authentic, and deepfakes of perceived trustworthy politicians are more likely to be regarded as authentic.

Finally, our search yielded one study which identified social factors which influenced how individuals perceived the credibility and authenticity of deepfakes. Jin et al. [75] presented participants with a YouTube video, where they manipulated the number of views the video had (representing the popularity of the video) and the number of followers the channel had (representing the trustworthiness of the source). They found that more views increased the perceived credibility of the video, and more followers increased perceived authenticity of the video. This study highlights how individuals have a tendency to perceive deepfakes as more credible and authentic if others appear to do so too. Once again though, the lack of studies on this topic limits the generalizability of these results. Notably, there were no studies exploring technical factors which influence perceptions of deepfakes, such as the quality or resolution of the deepfake, clarity of audio, visual artifacts, or realism of the deepfake.

#### RQ4. What kind of deepfake technology is being used in current empirical deepfake research?.

While not all the deepfakes used in the included studies were available for other researchers to view, it is clear that they varied considerably in terms of resolution, audio quality, accuracy of lip-syncing to voice, level of realism of the faces, and visual artifacts due to deepfake technology. As detailed in Table 1, they were taken from multiple different pre-made sources such as from the Canny AI art installation [4,56,60,64,70] and YouTube [4,36,71,75] or they were produced by the researcher themselves using software such as the ZAO mobile app [61], Faceswap software [57], custom made by a VFX artist or similar professional [58,66,67,74] or Tacotron 2 software [59]. The deepfakes included in this review that were drawn from the Canny AI art installation or YouTube could potentially be of a higher quality and more realistic since they were professionally produced by a team (Canny AI or Buzzfeed), but the downside to these studies is the lack of experimental control over the deepfakes used. The benefits for using pre-made deepfakes are that they enhance ecological validity as these deepfakes were made outside of an experimental setting, allow for comparison of results due to identical deepfake stimuli, and are freely available for other researchers to use to replicate the study allowing others to critically evaluate the work. Deepfakes which were produced by the researchers could be tailored towards answering specific research questions such as in Lee and Shin [57] or Dober et al. [59], where they manipulated specific faces or facial features and voices respectively rather than being constrained to designing experiments around pre-existing deepfakes. However, custom deepfakes give rise to issues of reporting as they are often not made readily available online [57,59]. This may be for ethical reasons due to researchers not wanting to contribute to the spread of misinformation online by uploading their custom deepfake, specifically if the deepfake was political in nature.

With deepfakes primarily being an audio-visual form of misinformation, their perceived credibility determines the quality of the deepfake. Only four studies from the 20 experimental studies in this review reported assessing the perceived credibility of the deepfake stimuli in their experiments as an indicator for quality [4,59,66,74], while six other studies assessed credibility as a dependent variable only [56,58,62,67,73,75]. Without assessing perceived credibility of deepfakes, it is difficult to interpret null results and assess video quality. Among the studies that reported assessing perceived credibility, Sharma et al. [74] asked a group of students (separate to the study sample) before the study to distinguish between an authentic video and a deepfake. Dobber et al. [59] and Murphy and Flynn [4] reported asking participants at the end of the study to rate the deepfakes they viewed on how authentic or credible the participant thought the deepfakes were, and Hameleers et al. [66] assessed credibility indirectly by comparing the perceived credibility and realism scores of statements made in authentic videos to deepfaked ones.

Of particular note, an overarching pattern seen across the four research questions is the short-term nature of the outcome measures in all of the studies included in this review. None of these studies included any kind of longer-term follow-up, instead all assessed beliefs, memories, or behavioural intentions instantly after the deepfake was viewed. Perhaps of more importance is the fact that most of the included studies did not include any comparator, so could not investigate what it is about deepfakes that may be novel or particularly efficacious at affecting beliefs, memories, and behaviours. The research indicates that while deepfakes may sometimes have a measurable impact on beliefs, memories, and behaviours, it is unclear if these effects are lasting or indeed any greater than effects of less technologically sophisticated forms of misinformation.

## Discussion

In total, our scoping review yielded 22 empirical papers that investigated the effects of viewing deepfakes, compared the deceptive ability of deepfakes to other misinformation mediums, or highlighted individual factors that are associated with vulnerability to deepfake effects. We found some evidence that deepfake exposure can manipulate beliefs about politicians and celebrities, enhance the credibility of misinformation messages, and induce false memories. However, the question of whether deepfakes are manipulative in a way that goes above and beyond existing forms of misinformation (such as text or doctored photos) is unclear, as many studies did not use experimental designs that allowed for such comparison. Finally, we found that cognitive ability is emerging as a factor that is associated with susceptibility to deepfake misinformation. Overall, while this review indicates that viewing deepfakes can bring about measurable effects, but incidences of conflicting findings and variety in study design and outcome measures used in those studies, means it is too early to draw concrete conclusions. We simply do not have enough evidence to support the widespread concerns that are often voiced in deepfake discourse [7,18,19,76].

To answer RQ1, we were interested in whether there was evidence of measurable effects of deepfakes on beliefs, memories, and behaviours. Eight papers found a measurable impact of deepfakes on beliefs, which broadly suggested that deepfakes may be able to enhance the vividness and credibility of misinformation messages, negatively impact attitudes towards political candidates, enhance mistrust towards real information and sources, enhance our self-esteem and perceived attractiveness, and negatively impact our views of deepfake resurrections. Two empirical studies found that deepfakes have a measurable impact on memories, capable of generating false memories for movies and entertainment videos. Finally, four studies identified a measurable effect of deepfakes on behavioural intentions, suggesting that deepfakes are more likely to be shared online due to their enhanced perceived credibility. However, these results are limited by the diversity of research topic, relatively low number of studies in each research area, and varying sources of deepfakes use in the experiments. We found that there is insufficient empirical data on the measurable effects of deepfake exposure to substantiate the circulating claims about deepfakes, such as jeopardizing national security, sabotaging political candidates, and undermining the foundation of truth in society [7,14,18].

Perhaps one of the largest gaps in this area of research is the lack of studies investigating any long-term effects of deepfake exposure on people’s beliefs, memories, and/or behaviours. This type of research is crucial in quantifying the effects of deepfake exposure because initial tests of the effects of seeing deepfakes may not detect significant shifts in attitudes or memories. This is because there could be a delayed “sleeper effect” in the misinformation. A “sleeper effect” can be defined as an increase in the influential power of a persuasive message after a period of time has passed, commonly associated with a discounting cue [77]. The theory behind a “sleeper effect” is that when individuals are exposed to a piece of misinformation, they will encode the message itself, as well as any reason to discount it, into their memory. Over a period of time, the original meaning behind the message is disassociated with the reasons to discount the message. Therefore, when the individual is exposed to the message again, they are more likely to solely recall the meaning behind the piece of misinformation and is more likely to be influenced by it without the discounting reasons [78]. Indeed, studies have found the presence of sleeper effects in memory and attitudinal studies ([78–80]). The potential presence of a “sleeper effect” suggests that deepfake exposure may have a significant effect on shifting beliefs, memories, or behaviours, but current deepfake research has not been designed to explore this question creating a knowledge gap.

Considering the wealth of speculation regarding the use of deepfakes in political environments and its threat to democratic processes [17–19], there is a clear disparity, though perhaps not a surprising one. Broinowski [81] has documented the history of alarmism at the advent of new technologies, from how telephones were speculated to eliminate privacy [82], to fears of a dystopian future with the arrival of the internet in the 1990s [82]. This alarmism typically spurs on a wealth of literature decrying new technologies, often neglecting to understand the people who utilise such technologies. Johnson and Verdicchio [83] describe a similar alarmism for AI technology as a phenomenon known as “sociotechnical blindness” where individuals fixate on the abstract concept of AI, rather than the human beings and social institutions utilising it. Omitting the human element behind new technologies such as deepfakes and focusing on the abstract threats and alarmist speculations could be a factor in the low number of empirical papers properly quantifying the harms of deepfakes to society and individuals. While our review calls for more empirical research on the harms of deepfake exposure, we also ultimately call for a more careful and evidence-focused approach to assessing the harms of new technologies in the future rather than blind speculation and alarmism. This sentiment has been echoed by some emerging literature decrying technological alarmism [82].

For RQ2, we wanted to see if there was evidence that compares the effects of deepfakes with pre-existing forms of misinformation to identify whether deepfakes are uniquely persuasive and deceptive. We only found six studies which provided evidence for RQ2, and they suggest that deepfakes may not be uniquely effective at distorting memories compared to pre-existing forms of misinformation, but the evidence is mixed when assessing whether deepfakes enhance the credibility of misinformation messages more than pre-existing formats. Only finding six studies with comparators to other misinformation formats is surprising, as the headline results of the other empirical studies are often that deepfakes can manipulate us in some way, perhaps implying that the identified form of manipulation is unique to deepfake technology. It is imperative that researchers clarify the unique position of deepfakes in misinformation literature and we strongly recommend that future experiments include a less technically advanced comparator, such as simple text-based misinformation. This would help establish whether deepfakes should be treated as just another form of misinformation or whether they are indeed a special case [8,36,84].

In RQ3, we were interested in any evidence which outlines psychological, social, or technical factors which influence the effect of deepfakes on beliefs, memories, and behaviours. We found 13 studies which explored how psychological and social factors such as cognitive ability, message congruency, and affect individuals’ beliefs memories, and behaviours. Results from these studies broadly suggest that individuals with higher cognitive ability are less likely to consider deepfakes as credible and share them, while individuals who agree with the message behind misinformation presented as deepfakes are more likely to believe and share them. However, these results are obfuscated by poor methodology and therefore are difficult to draw conclusions from. Despite this, our findings on cognitive ability reflect results from broader misinformation literature, i.e., cognitive ability is associated with reduced susceptibility to deepfakes. This has been demonstrated in existing research concerning misinformation in the form of news headlines, social media posts, or other text-based formats [5,50,85]. Some of the studies included in this review suggest that deepfake-based misinformation is especially potent in spreading misinformation due to the multi-modal aspect of deepfakes enhancing the vividness and credibility of the misinformation [56,57,65], but other studies dispute this [4,63,66,67]. The lack of comparative studies assessing the predictive power of cognitive ability for misinformation susceptibility in different formats means that we cannot deduce if there is anything special about deepfakes in this regard, or if these findings are merely an extension of what is already established as a predictive factor in misinformation susceptibility.

We were interested in identifying the types of deepfakes being used in deepfake empirical papers for RQ4. The experiments included in this review used deepfakes of varying quality from different sources, including Tacotron 2 and AI based lip synchronization techniques, Canny AI art installation, Faceswap software, ZAO deepfake app, and publicly available deepfakes taken from YouTube, Reddit and other internet sites. Many studies did not share the deepfakes used in the experiment, making it difficult to compare the quality of materials between studies, though we did note that some of the deepfakes that were accessible were of quite poor quality. Little research has been done to identify the effect of the “quality” of a deepfake on its effectiveness to influence beliefs, memories, and/or behaviours. In fact, there is little consensus on what a “high quality” deepfake is [86]. It is possible that the quality of a deepfake significantly affects its influential ability as some research has demonstrated that the perception of deepfakes depends on its realism and human-likeness [87]. Despite this gap in literature, we do not advocate for the standardisation of deepfake quality in empirical research because researchers may choose specific software/deepfake stimuli for different reasons (accessibility, financial constraints, resource and time constraints) thus pushing for a standardized quality may act as a barrier restricting empirical deepfake research. We do however suggest checking the perceived credibility of deepfake stimuli in future experimental research. This allows future researchers to identify whether the deepfakes were of a sufficient quality to successfully influence participants. We suggest pilot testing of the deepfake stimuli to assess their rate of identification compared to authentic videos before conducting the study and clear reporting of measures of video quality in the write-up.

Our findings suggest that despite widespread claims about the ease of access to deepfake technology [7,20,88], researchers in this area clearly find it difficult to create custom high-quality, realistic deepfakes. This may be due to a lack of time, computing infrastructure, or expertise, but the end result is that very few researchers have used custom-created high-quality deepfakes in their studies [58,66,67]. Thus, our results may not reflect the true manipulative efficacy of high-quality deepfakes that may become more accessible in the future. Where researchers cannot create their own high-quality deepfakes, some have used existing YouTube videos [4], while others have resorted to asking participants to imagine hypothetical deepfakes [89]. Neither solution is without its drawbacks. Using existing videos incurs the risk that participants will have seen the video before, and the studies included in this review differed in whether they removed participants who had previously seen the video [4] or opted to retain them in the analyses [36]. Asking participants to imagine a deepfake is clearly a difficult task and participants’ ability to do so will be hugely affected by their technical literacy [89]. For these reasons, we strongly encourage more collaboration between psychologists who are interested in testing the cognitive effects of deepfake exposure and those with more technical expertise who can build convincing custom deepfakes for use in these studies. We also encourage researchers to make their materials available to other researchers where possible, so that the quality of the deepfakes can be assessed. Only by establishing more rigorous and transparent methodologies will we gain insight into the effects of deepfakes.

Through reviewing existing research on the topic, we identified a number of methodological limitations that impede our ability to draw strong conclusions from the existing data. Firstly, while some studies in our review have included behavioural measures such as sharing intentions [56,65,68,74], there is little empirical deepfake research exploring other real-world behavioural consequences of deepfakes such as voting intentions or intentions to vaccinate. Our review highlights an overall lack of research that investigates the effect of viewing deepfakes on people’s behaviour. This finding is not unique to deepfake research – a recent review of misinformation research found that only 29% of empirical misinformation studies between the years 2016–2022 examined the impact of misinformation on behaviours or behavioural intentions [90]. Most of those studies only examined online behaviours or behavioural intentions (e.g., intent to share a news story on social media), so that only 11% of studies examined offline behaviours or behavioural intentions (e.g., intent to get vaccinated). We echo the call by Murphy et al. [90] and encourage researchers to examine behavioural effects of deepfakes, where we currently have little evidence but rampant speculation [18,47,76]. Of course, research in this area will need to follow best practice in terms of ethical practices, including effective debriefing of participants as soon as possible [91].

While we have highlighted a tendency of existing research to overlook the need for empirical literature in favour of alarmist speculations, we feel the need to also highlight the current real-world harms of deepfakes, and interestingly, the lack of empirical deepfake literature underpinning these real-world applications. An application of deepfakes that did not appear in the scoping review is pornography. Estimates suggest that 96% of existing online deepfakes are pornographic in nature [19], it is therefore of note that there were no empirical studies quantifying the effects of deepfake porn exposure in the scoping review (though our search terms did not seek these studies out). Deepfake pornography can potentially pose a threat to the depicted person’s reputation and mental health through its dissemination and subsequent distress, similar to how non-consensual spread of intimate images online can cause emotional distress [23,24]. Deepfake pornography may also pose threats to consumers, similar to how general pornography can negatively “shape sexual scripts” and influence how consumers behave during sexual encounters [92]. Legislation and research are converging on deepfake pornography in an attempt to understand and regulate its impacts through policy [7,15,24,93]. Rigorous, ethical research that gives us a clearer understanding of the effects of deepfake pornography is urgently required, as we attempt to develop effective policies and countermeasures.

There are several limitations to the current study. Firstly, due to the different interpretations and definitions of deepfakes in deepfake literature [53], it is possible that some studies used deepfakes in an experimental manner but failed to describe them with the term “deepfake”. While we took all reasonable steps to include these studies in our review, it is possible that we could have missed them. Secondly, we did not include any preprints or conference abstracts in our search. This study is a scoping review of peer-reviewed literature on the topic of deepfake effects on beliefs, memories and behaviours and therefore those elements were omitted. However, it is possible that informative research related to our research questions could be found in preprints and conference abstracts, and therefore some information related to our research topic could have been missed.

In conclusion, the current scoping review maps the current state of empirical deepfake literature and provides specific suggestions for future empirical deepfake research. The most obvious suggestion is for an overall increase in empirical deepfake papers quantifying the effects of deepfake exposure first before speculation. We also advocate for more empirical deepfake studies to test the potential longitudinal effects of deepfake exposure, to test a broader range of behavioural implications of deepfake exposure and include comparators to deepfakes in experiments such as text or audio misinformation to investigate the unique effectiveness of deepfakes. While research in this field is clearly still in its infancy, this review highlights some persistent methodological issues as well. In general, we conclude that there are a great many papers decrying the risks and harms of deepfakes without the necessary empirical evidence to support these alarmist claims. We cannot hope to design interventions or implement regulations to combat these concerns unless we first quantify the issues. Therefore, we call for further (methodologically rigorous) research on the effects of deepfake exposure.

## Supporting information

S1 Checklist PRISMA-ScR fillable checklist_11Sept2019 (1). (PDF)
